# Prevalence and molecular heterogeneity of *Bartonella bovis* in cattle and *Haemaphysalis bispinosa* ticks in Peninsular Malaysia

**DOI:** 10.1186/s12917-015-0470-1

**Published:** 2015-07-16

**Authors:** Kai-Ling Kho, Fui-Xian Koh, Tariq Jaafar, Quaza Nizamuddin Hassan Nizam, Sun-Tee Tay

**Affiliations:** Department of Medical Microbiology, Faculty of Medicine, University of Malaya, 50603 Kuala Lumpur, Malaysia; Department of Veterinary Services, Ministry of Agriculture and Agro-Based Industry Malaysia, Federal Government Administrative Centre, 62630 Putrajaya, Malaysia

**Keywords:** *Bartonella bovis*, Cattle, *Haemaphysalis bispinosa*, Ticks

## Abstract

**Background:**

Bartonellosis is an emerging zoonotic infection responsible for a variety of clinical syndromes in humans and animals. Members of the genus *Bartonella* exhibit high degrees of genetic diversity and ecologic plasticity. The infection is usually transmitted to animals and humans through blood-feeding arthropod vectors such as fleas, lice, ticks and sandflies. This study was conducted to investigate the prevalence of *Bartonella* species in 184 beef cattle, 40 dairy cattle, 40 sheep and 40 goats in eight animal farms across Peninsular Malaysia. *Bartonella*-specific PCR assays and sequence analysis of partial fragments of the citrate synthase gene were used for detection and identification of *B. bovis*. Isolation of *B. bovis* was attempted from PCR-positive blood samples. Molecular heterogeneity of the isolates was investigated based on sequence analysis of *gltA*, ITS, *rpoB* genes, ERIC-PCR, as well as using an established multilocus sequence typing (MLST) method. The carriage rate of *B. bovis* in ticks was also determined in this study.

**Results:**

*B. bovis* was detected using *Bartonella gltA*-PCR assays from ten (4.5 %) of 224 cattle blood samples, of which three (1.3 %) were from beef cattle and seven (3.1 %) were from dairy cattle. None of the blood samples from the sheep and goats understudied were positive for *B. bovis. Haemaphysalis bispinosa* and *Rhipicephalus (Boophilus) microplus* were the predominant tick species identified in this study. *B. bovis* was detected from eight of 200 *H. bispinosa* ticks and none from the *R. microplus* ticks. Isolation of *B. bovis* was successful from all PCR-positive cattle blood samples, except one. Strain differentiation of *B. bovis* isolates was attempted based on sequence analysis of *gltA*, ITS, *rpoB*, and ERIC-PCR assay. *B. bovis* isolates were differentiated into six genotypes using the approach. The genetic heterogeneity of the isolates was confirmed using MLST method. Of the six MLST sequence types identified, five were designated new sequence types (ST23-27), while one (ST18) had been reported previously from Thailand isolates. All except one isolates were segregated into lineage II. A new lineage (IIa) is proposed for a single isolate obtained from a dairy cow.

**Conclusions:**

The current study reported the first detection of *B. bovis* infection in the cattle and *H. bispinosa* ticks in Peninsular Malaysia. At least six genotypes of *B. bovis* were found circulating in the cattle understudied. New MLST sequence types were identified in Malaysian *B. bovis* isolates. Further study is necessary to explore the zoonotic potential of *B. bovis* and the vector compatibility of *H. bispinosa* ticks.

## Background

Bartonellae are small, Gram-negative fastidious bacteria which infect mammalian erythrocytes and endothelial cells. The organisms have been reported as the causative agents for a variety of clinical symptoms in human and animals [1, 2]. Transmission of the bacteria is usually through the bites of hematophagous arthropods such as fleas, sand flies, biting flies, lice and ticks [3–6]. Various bartonellae species have been associated with domestic and wild ruminants. The infections caused by bartonellae in the cattle are usually asymptomatic. *Bartonella bovis*, previously known as *Bartonella weissii* and originally isolated from domestic cats, has been reported as a pathogen which causes endocarditis and bacteremia in cattle [7–16].

The prevalence of *B. bovis* in cattle varied tremendously in different studies: USA (50–89 %), French Guyana (70 %), Georgia (57 %), Taiwan (42.4 %), France (36 %), Italy (24 %), Guatemala (21 %), West Africa (20 %), Thailand (10 %), Poland (6.8 %), Japan (0 %) and Kenya (0 %) [8–16]. Cattle ticks (*Rhipicephalus microplus*) and flies have been described as the potential vectors for transmission of *B. bovis* [3, 15]. In a recent study, multiple factors such as distribution and abundance of specific arthropods, and environmental factors (geographic characters, landscape, etc.) have been postulated to have some effect on the prevalence of *B. bovis* [14]. Other than the isolation of *B. melophagi* from sheep ked, little is known about the prevalence of *Bartonella* spp. in sheep and goats [17].

Information on the prevalence, genetic variation and arthropod vector is important for formulation of strategies for prevention and control of *B. bovis* infections. Several molecular approaches including sequence analysis of the *Bartonella* citrate synthase gene (*gltA*) [18], internal transcribed spacer of 12S-23S rRNA (ITS) [19], and beta subunit of the RNA polymerase gene (*rpoB*) fragments [20], enterobacterial repetitive intergenic consensus (ERIC)-PCR [21], and PCR-restriction fragment length polymorphism [18] have been used for genotyping of bartonellae. Recently, a multi-locus sequence typing (MLST) scheme has been developed to compare the genetic divergence of *B. bovis* [14]. The MLST data suggested genetic variations among 28 isolates from different geographical regions and a total of 22 sequence types and three lineages (I, II, and III) had been identified.

Data on the prevalence and genetic diversity of *B. bovis* in the domestic and wild animals in the Southeast Asia is scarce. So far, *B. bovis* infections have only been reported from cattle and water buffaloes from Thailand [14]. There has not been any report on the association of *B. bovis* with any arthropod in the region. This study was designed to determine the prevalence and molecular heterogeneity of *Bartonella* spp. in the cattle, sheep and goats from eight farms across Peninsular Malaysia and to investigate the presence of bartonellae in the ticks collected from the farms. Isolation and strain differentiation of *B. bovis* were also attempted.

## Methods

### Study sites and sample collection

Written approval (JPV/PSTT/100-8/1) for animal blood sampling and assessment of tick samples was obtained from the Director, Department of Veterinary Services, Ministry of Agriculture and Agro-Based Industry, Malaysia who owns the animals understudied. Sampling was carried out by farm workers according to standard veterinary care and practice. Table 1 shows the locations of the farms selected in this study. Animal blood sampling was conducted from February to September 2013 in six cattle farms, a goat and a sheep farm at different regions of Peninsular Malaysia. Approximately 1–3 mL whole blood samples were collected from the animals via jugular vein in EDTA-coated tubes and transported on ice to the laboratory. Blood samples were stored at -20 °C prior to processing.

**Table 1:** Detection of *B. bovis* using *gltA* PCR assays in cattle, sheep and tick samples collected from eight farms in Peninsular Malaysia

States	Farms (GPS location)	Date of sample collection	Animal breed (n)	No. blood sample collected	No. (%) PCR-positive blood sample	No. of animal infested with ticks (No. and ticks species subjected to PCR screening)	No. (%) PCR-positive ticks
Negeri Sembilan	Beef cattle farm 1	February 2013	Nellore (11), YKK (15)	26	1 (3.8)	27 (6 *H. bispinosa* and 41 *R. microplus*)	4 (8.5)
	(N2° 40′ 2.273′′ E102 ^o^ 34′ 23.022′′)						
Pahang	Beef cattle farm 2	August 2013	Kedah-Kelantan (Zebu) (38)	38	0 (0)	9 (14 *R. microplus*)	0 (0)
	(N3° 56′ 48.61′′ E102 ^o^ 22′ 47.57′′)						
	Beef cattle farm 3	August 2013	Nellore (32), Brahman (8)	40	0 (0)	38 (37 *R. microplus* and 57 *H. bispinosa*)	0 (0)
	(N3° 45′ 24.059′′ E103 ^o^ 12′ 12.92′′)						
Kedah	Sheep farm 4	September 2013	Damara (40)	40	0 (0)	21 (44 *H. bispinosa*)	0 (0)
	(N6°9′ 13.478′′ E100 ^o^ 32′ 3.379′′)						
Kelantan	Beef cattle farm 5	September 2013	Kedah-Kelantan (40)	40	0 (0)	Not determined (0)	0 (0)
	(N5° 51′ 48.45′′ E102 ^o^ 0′ 52.97′′)						
Terengganu	Beef cattle farm 6	September 2013	YKK (40)	40	2 (5.0)	39 (13 *R. microplus* and 58 *H. bispinosa*)	4 (5.6)
	(N5° 4′ 8.926′′ E102 ^o^ 59′ 49.45′′)						
Negeri Sembilan	Goat farm 7	September 2013	Boer (32), Savannah (2), African dwarf (5), Cashmere (1)	40	0 (0)	0 (0)	0 (0)
	(N2° 56′ 49.75′′ E102 ^o^ 5′ 7.635′′)						
Johore	Dairy cattle farm 8	September 2013	Jersey (4), Mafriwal (36)	40	7 (17.5)	0 (0)	0 (0)
	(N2° 1′ 43.63′′ E103 ^o^ 18′ 55.36′′)						
			Total:	304	10 (3.3)	134 (270)	8 (3.0)

All the cattle and sheep farms were managed by rotational grazing system while goat farm 7 was kept under zero grazing practice

*YKK* Yellow cattle cross Kedah-Kelantan

Ticks were collected from animals subjected for blood collection whenever possible. The ear, eyes, flank, abdomen, tail and perineal regions of the animals were examined for ticks. Ticks were identified to the genus level according to Walker *et al.* [22] and Geevarghese and Mishra [23], and preserved in -80 °C freezer prior to processing. For molecular identification of the ticks, the tick 16S rRNA gene was amplified and sequenced [24].

### DNA extraction

DNA was extracted from 200 μL of animal blood samples using QIAamp DNA mini kit (Qiagen, Hilden, Germany) according to the manufacturer’s instructions. Ticks were first thawed, washed in 5 % sodium hypochlorite and 70 % ethanol and rinsed in sterile distilled water prior to homogenization using surgical blades [25]. Each tick homogenate was then subjected to DNA extraction using a QIAamp DNA mini kit (Qiagen, Hilden, Germany).

### Molecular detection and data analysis of *Bartonella* spp. from animal blood samples and tick samples

PCR assay targeting bartonellae citrate synthase gene (*gltA*) was used for detection of *Bartonella* DNA in the animal blood and tick samples [18]. All PCR assays were performed in a final volume of 20 μL containing 2 μL of DNA template, 1X ExPrime *Taq* DNA polymerase (GENET BIO, South Korea) and 0.2 μM of each primer, in a Veriti thermal cycler (Applied Biosystems, USA). Positive control derived from plasmid carrying the amplified *gltA* gene from *Bartonella tribocorum* and negative control (sterile distilled water) were included in each PCR run. A rodent-borne strain of *Bartonella elizabethae* (BeUM) was used as positive control for other PCR assays. PCR products were separated on a 1.5 % agarose gel at 100 V for 45 min and visualized using a UV transilluminator (G-Box, Syngene, UK). Amplicons were purified using GeneAll Expin™ Combo GP (GeneAll, South Korea) as described by the manufacturer. Sequencing was performed with a Big Dye Terminator Cycle Sequencing kit, version 3.1 (Applied Biosystems, USA) on an ABI PRISM 377 Genetic Analyzer (Applied Biosystems, USA), using forward and reverse primers.

Sequence assembly and alignment were performed using BioEdit Sequence Alignment Editor Software (Version 7.0.5.3). The resulting sequences were compared with known *Bartonella* sequences deposited in the GenBank database using the Basic Local Alignment Search Tool (BLAST) program (http://blast.ncbi.nlm.nih.gov/Blast.cgi).

### Isolation of *Bartonella* spp

Isolation of *B. bovis* was attempted using PCR-positive blood samples. Each blood sample (100 μL) was inoculated in duplicate onto fresh Columbia agar plates supplemented with 5 % sheep blood (Isolab, Malaysia). The agar plates were incubated at 37 °C in 5 % CO_2_ for four weeks. Following incubation, isolates were subcultured for Gram staining, and DNA was extracted using a QIAamp DNA mini kit (Qiagen, Hilden, Germany). Confirmation of the identity of the isolates was carried out by using *gltA*-PCR followed by sequence analysis.

### Strain differentiation *B. bovis* isolates

*B. bovis* isolates were differentiated based on sequence analysis of *gltA* (278 bp) [18], ITS (301–322 bp) [19], *rpoB* (671 bp) [20], and ERIC-PCR assay [21]. Enterobacterial repetitive intergenic consensus (ERIC)-PCR was performed by using ERIC 1R and ERIC 2 primers as described previously [26]. The PCR reaction mixture contained 2 μL of DNA template, 0.2 μM of each primer, 1 X PCR buffer, 0.2 mM dNTP mix, 1.5 mM magnesium chloride (MgCl_2_) and 1U of Go*Taq*^@^ Flexi DNA polymerase (Promega Corp, USA). Amplification was performed according to Dehio *et al.* [21] and the amplified fragments were separated by electrophoresis at 60 V in a 2 % agarose gel for 4 h.

### MLST analysis

Sequence comparison for eight loci (16S rRNA, *ftsZ*, *groEL*, *nuoG*, *ribC*, *rpoB*, *ssrA* and ITS) were performed for each *B. bovis* isolate as described by Bai *et al.* [14]. A dendrogram was constructed based on the concatenated sequences of the eight loci using the neighbour-joining method of the MEGA 6.0 software and bootstrap analysis with 1,000 resamplings [27].

## Results

A total of 304 blood samples collected from 184 beef cattle, 40 dairy cattle, 40 sheep and 40 goats were subjected to PCR detection for *B. bovis* DNA in this study (Table 1). The prevalence of *B. bovis* ranged from zero to 17.5 % across the eight animal farms. *B. bovis* was detected from ten (10/224; 4.5 %) of 224 cattle blood samples, of which three (3/224; 1.3 %) were from beef cattle and seven (7/224; 3.1 %) were from dairy cattle. None of the blood samples from the sheep and goats understudied were positive for *B. bovis*. The PCR-positive blood samples were derived from three beef cattle (one Nellore and two Yellow cattle cross Kedah-Kelantan breeds) in two farms (Farms 1 and 6) and seven dairy cattle (Mafriwal breed) in Farm 8 (Table 1). The age of the infected cattle ranged from one to four years old. No overt clinical signs were observed from those cattle with positive PCR findings for *B. bovis*.

Mixed populations of *R. microplus* and *H. bispinosa* were detected in the cattle in this study. The sheep were mainly infested by *H. bispinosa*. Of 270 ticks examined in this study, 70 and 200 ticks were identified as *R. microplus* and *H. bispinosa*, respectively. Sequence analysis of the 16S rRNA gene amplified from *B. bovis*-positive ticks revealed 100 % identity (200 bp) with that of *H. bispinosa* reported in India (GenBank: KC853418- KC853420) [28]. Eight (4.0 %) *H. bispinosa* ticks collected from *B. bovis-*infected cattle in two farms (Farms 1 and 6) were positive using *Bartonella gltA* PCR assay. These included one male tick and seven fully engorged female ticks. None of the *R. microplus* tick was positive for bartonellae.

*B. bovis* was successfully isolated from nine cattle blood samples in this study (Table 2). A total of one, two and six isolates were cultured from the blood samples collected from cattle in Farms 1, 6 and 8, respectively. Primary cultures showed the growth of tiny, discrete, and greyish colonies after 5–7 days of incubation. A low level of bacteremia (≈20 colony forming unit/mL) was suspected as only one to two single colonies were obtained from each blood sample. The isolates were confirmed as *B. bovis* by using *gltA*-PCR assay followed by sequence analysis.

**Table 2:** Genotyping of nine *B. bovis* isolates using a combined sequence analysis of *gltA*, ITS, *rpoB* and ERIC-PCR assay

Sample ID	Farm (age, sex, breed)	*gltA*	ITS	*rpoB*	ERIC-PCR	Genotype
		(278 bp)	(301 bp & 322 bp)	(671 bp)	profile	
F1-1	1(2 years, M, Nellore)	B1	S2	R2	E2	1
F6-1	6 (1 year, F, YKK)	B1	S3	R1	E1	3
F6-2	6 (1 year, M, YKK)	B1	S1	R1	E1	2
F8-1	8 (1 year, M, Mafriwal)	B1	S2	R2	E2	1
F8-2	8 (1 year, F, Mafriwal)	B1	S3	R1	E1	3
F8-3	8 (1 year, F, Mafriwal)	B1	S3	R1	E1	3
F8-4	8 (3 years, F, Mafriwal)	B1	S1	R4	E1	4
F8-5	8 (1 year, M, Mafriwal)	B1	S1	R3	E1	5
F8-6	8 (1 year, M, Mafriwal)	B2	S1	R5	E3	6

*M* Male, *F* Female, *YKK* Yellow cattle cross Kedah Kelantan

The isolates were first differentiated based on the nucleotide variation of *gltA*, ITS, *rpoB* and ERIC-PCR assay (Table 2). The sequences derived from this study have been deposited in the GenBank database: B1-B2 (*gltA*, GenBank accession no.: KP230412-KP230413), S1-S3 (ITS, GenBank accession no.: KP230415-KP230417), and R1-R5 (*rpoB*, GenBank accession no.: KP230418-KP230422). Two *gltA* sequence types (B1-B2), demonstrating 100 % (n = 8) and 99 % (n = 1) similarities with *B. bovis* strain 91-4 (GenBank accession no.: AF293394), were identified in this study. The amplified ITS gene from nine isolates was differentiated into three sequence types (S1-S3). The 322 bp of the sequence type S1 (five isolates) and S2 (two isolates) showed 99 % homology with *B. bovis* strain 91-4 (GenBank accession no.: AY116638). Meanwhile, the 301 bp of the sequence type S3 were aligned 100 % (292/292) to that of *B. bovis* strain B38216 from the cattle in Guatemala (GenBank accession no.: KF218233, [14]). The *rpoB* sequences obtained from nine isolates were differentiated into five sequence types (R1-R5). Sequence type R1 was identical to that of *B. bovis* strain B25099 from cattle in Thailand (GenBank accession no.: KF218220) [14]. Sequence type R2, R3 and R4 showed the closest similarity (99 %) to that of *B. bovis* strain B32674 from a water buffalo in Thailand (GenBank accession no.: KF218223) [14]. The sequence type R5 identified in an isolate (F8-6) in Farm 8 was identical to that of a *B. bovis* isolate (N04-927) which caused bovine endocarditis in France (GenBank accession no.: EF432062) [8].

**Fig. 1:**
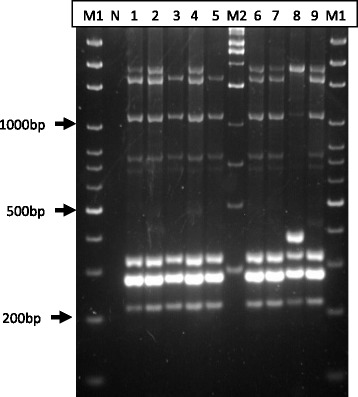
ERIC-PCR fingerprinting profiles of nine *B. bovis* isolates. Lane M1, 100 bp ladder; Lane M2,1 kb ladder; Lane 1: F6-1 (E1); Lane 2: F6-2 (E1); Lane 3: F1-1 (E2); Lane 4: F8-4(E1); Lane 5: F8-1 (E2); Lane 6: F8-2 (E1); Lane 7: F8-3 (E1); Lane 8: F8-6 (E3) and Lane 9: F8-5 (E1)

Figure 1 shows the fingerprinting profiles generated from the ERIC-PCR assay. A total of four to seven DNA fragments with the size ranging from 210 bp to 2 kb were generated from each isolate. Based on the fingerprinting profiles in Fig. 1, the isolates were differentiated into three genotypes (E1-E3). Table 2 summarizes the genotyping results of *B. bovis* isolates based on the combined sequence analysis of *gltA*, ITS, *rpoB* and ERIC-PCR assay. A total of six genotypes of *B. bovis* were identified from nine isolates in this study. Interestingly, six isolates from a single farm (Farm 8) were differentiated to five genotypes (Table 2, Fig. 1). The approach grouped the isolates F1-1 and F8-1 in Genotype 1, and the isolates F6-1, F8-2 and F8-3 in Genotype 3. The remaining isolates, i.e., F6-2, F8-4, F8-5 and F8-6, were differentiated to Genotype 2, 4, 5 and 6, respectively. Further differentiation of *B. bovis* from blood and tick samples based on the sequence analysis of multiple genes was not possible due to the failure in amplifying the target genes, probably owing to the low amounts of DNA in the samples.

Six sequence types were identified from the MLST analysis in this study (Table 3), of which five (designated as ST23-27) have not been described before. New sequences obtained from the MLST analysis in this study have been submitted to the GenBank database with the accession numbers KR733181, KR733182-KR733184, KR733185-KR733186, KR733187-KR733189, KR733190-KR733191, KR733192-KR733195, KR733196, KR733197-KR733121 for *ftsZ*, *gltA*, *groEL*, *nuoG*, *ribC*, *rpoB*, *ssrA* and ITS genes, respectively.

**Table 3:** Allelic profiles, sequence type (ST), and lineage group (LG) for nine *B. bovis* isolates as determined using MLST approach

Isolate	*ftsZ*	*gltA*	*groEL*	*nuoG*	*ribC*	*rpoB*	*ssrA*	ITS	ST	LG
F1-1(Y75)	2	7^a^	5	6^a^	9^a^	10^a^	6^a^	13^a^	23	II
F6-1 (1789)	2	2	5	2	2	4	2	4	18	II
F6-2 (1960)	2	8^a^	13^a^	5^a^	3	4	2	9^a^	24	II
F8-1 (I82 4622)	2	7^a^	5	6^a^	9^a^	10^a^	6^a^	13^a^	23	II
F8-2 (I82 4644)	2	2	5	2	2	4	2	4	18	II
F8-3 (I82 4648)	2	2	5	2	2	4	2	4	18	II
F8-4 (F5X4371)	2	8^a^	5	5^a^	3	12^a^	2	11^a^	25	II
F8-5 (I82 4623)	2	8^a^	5	5^a^	3	11^a^	2	10^a^	26	II
F8-6 (I72 4598)	8^a^	6^a^	14^a^	7^a^	10^a^	9^a^	2	12^a^	27	IIa

^a^New allelic profile

**Fig. 2:**
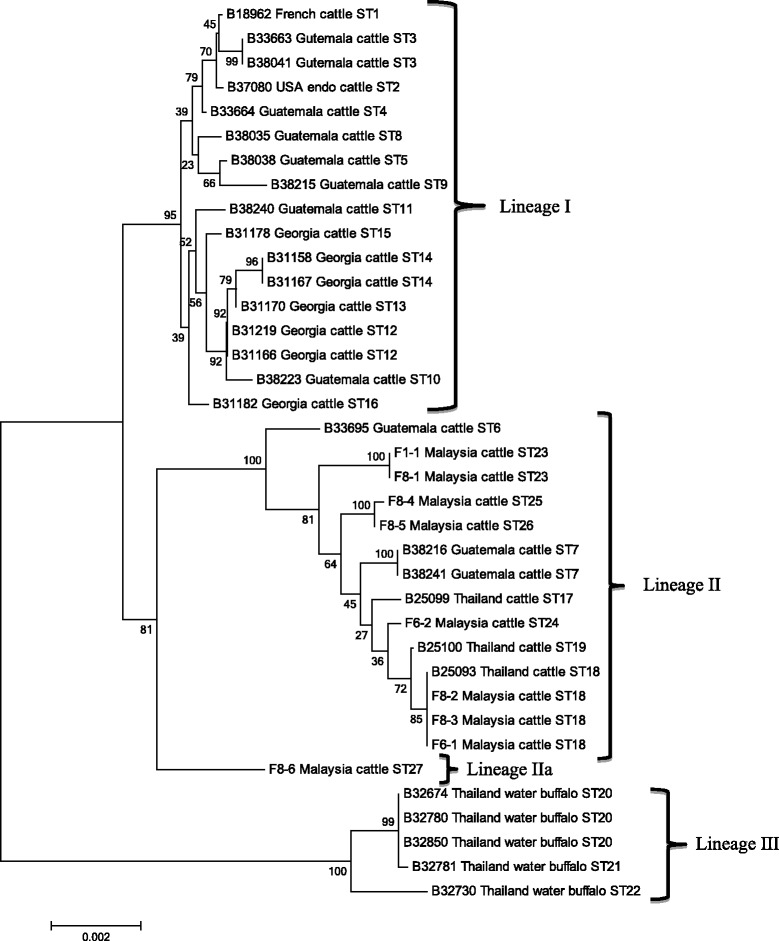
Phylogenetic placement of Malaysian *B. bovis* isolates based on the concatenated sequences of *ftsZ*, *gltA*, *groEL*, *nuoG*, *ribC*, *rpoB*, *ssrA*, and ITS genes. Reference sequences were retrieved from Bai *et al.* [14] for comparison. Bootstrap analysis was performed with 1000 replications. Numbers in brackets are GenBank accession numbers. Scale bar indicates the nucleotide substitutions per sites

Three isolates (F8-2, F8-3 and F6-1) in this study were identified as ST18 which had been reported in *B. bovis* isolated from cattle in Thailand (B25093). ST23 was represented by two isolates (F1-1 and F8-1) in this study. Other STs (ST24-27) in this study were represented by a single isolate. One of the isolates (F8-6) with unique PCR fingerprint profile (E3) exhibited sequence variation in all the gene fragments except for *ssrA* gene. The dendrogram constructed based on the concatenated sequences of eight loci demonstrated the segregation of eight *B. bovis* isolates into lineage II (Fig. 2). The isolate F8-6 (ST27) was placed at a single branch adjacent to those isolates from the lineage II, with a 81 % bootstrap value. The isolate is proposed under a new lineage (designated as lineage IIa).

## Discussion

This study reports for the first time the molecular detection and isolation of *B. bovis* from cattle in Peninsular Malaysia. The prevalence (4.5 %) of the *B. bovis* in the cattle, as determined by direct amplification from blood samples in this study was low when compared to those of cattle (10 %) and water buffaloes (6.8 %) reported from Thailand, a neighbouring country of Malaysia [14]. A high prevalence (42.4 %) of *B. bovis* has been reported in beef cattle from Taiwan [15]. In this study, the prevalence of *B. bovis* infections was higher (7/40; 17.5 %) in dairy cattle, as compared to the beef cattle (3/204; 1.6 %). In a Japanese study, *B. bovis* was not detected from 305 cattle investigated across five prefectures [14]. The causes for the variation in the prevalence of *B. bovis* across the same geographical region are still not clear [14]. Factors such as the carriage rate of *B. bovis* in the ectoparasites, environmental exposure, animal breed, susceptibilities and husbandry practices are probably important. Although cattle examined in this study appeared healthy, *B. bovis* infection has been associated with endocarditis in previous investigations. Maillard *et al.* reported *B. bovis* infection in 9.1 % of 22 cows with endocarditis [7]. Additionally, *B. bovis* had been isolated from the heart of an Angus cow which succumbed to sudden death without any clinical sign [29]. As endocarditis caused by fastidious haemotropic bacteria is not frequently accompanied by clinical manifestations [30], direct amplification of *B. bovis* DNA from blood samples by using PCR method is important for surveillance of the infection in the animals.

*R. microplus* is a common tick in cattle while *H. bispinosa* tick has been reported to parasitize both cattle and goats [28]. *B. bovis* has been detected from 15.7 % of *R. microplus* ticks in Taiwan [15]. The cattle in this study were infested by both *R. microplus* and *H. bispinosa*, while the sheep were infested mainly by *H. bispinosa*. Although Farm 8 had the highest prevalence of *B. bovis*, ticks were not available for testing as the animals had undergone deticking prior to our sampling study. To the best of our knowledge, this is the first report of the detection of *B. bovis* in *H. bispinosa* ticks. *Haemaphysalis* genus is the second largest tick genus in the family *Ixodidae* [31] which has been associated with various tickborne pathogens including *Bartonella* [32], *Theileria*, *Babesia*, *Rickettsia*, *Ehrlichia*, *Anaplasma* [33], and spotted fever group rickettsiae [34]. *Bartonella* spp. other than *B. bovis* have been reported in *H. longicornis* in ticks from Korea [32] and China [35].

In this study, although *B. bovis* has been detected from both infected cattle (F6-2) and *H. bispinosa* ticks feeding on the same host, this does not necessarily demonstrate *B. bovis* infection in the ticks as the DNA detected might have originated from the blood ingested by ticks. Further characterization and experimental transmission studies are needed to demonstrate vector competency of the *H. bispinosa* for *B. bovis* transmission.

Sequence analysis of *gltA*, ITS and *rpoB* genes and ERIC-PCR assay was initially attempted for strain differentiation of *B. bovis* in this study. Among the three genes, most sequence variation was observed in the *rpoB* gene. Sequence analysis of *gltA* and ITS region were less discriminative due to their shorter fragments. ERIC-PCR assay was rapid and cost-effective but less discriminative compared to the results obtained based on the sequence analysis of the *rpoB* gene. However, when the results of the sequence typing of *gltA*, ITS, *rpoB* and ERIC-PCR were combined (as shown in Table 2), six genotypes were identified in the nine isolates. Although the discrimination power of this approach was similar with MLST analysis, further validation with more isolates is necessary.

In an effort to reveal the genetic diversity of *B. bovis,* Bai *et al.* reported the segregation of 22 STs into three lineages (I-III) from 28 strains originated from different continents [14]. The typing of nine *B. bovis* isolates understudied into six STs confirms the high genetic diversity of *B. bovis*, particularly for those isolated from dairy cattle in Farm 8, with the identification of five STs (Table 2). Specific host association had been demonstrated previously with isolates from lineages I and II in cattle and lineage III in water buffaloes [14]. It was hypothesized that lineage I was associated with the cattle of ‘taurine’ lineage while lineage II was associated with the cattle of ‘zebu’ lineage. The crossing lineages of *B. bovis* isolated from the cattle from Guatemala (lineage I and II) had been attributed to a mixed breed of cattle. Interestingly, in this study, *B. bovis* was isolated with a higher rate in the dairy cattle (Mafriwal breed). The tropical breed has been developed by crossbreeding between Sahiwal (*Bos indicus*) and Friesian (*Bos taurus*) cattle to gain higher weight and milk production [36]. The finding of new STs and proposal of a new lineage for *B. bovis* isolated from the crossbreed cattle are interesting and warrants further investigation.

## Conclusions

The detection of *B. bovis* in cattle blood and *H. bispinosa* tick samples from six farms across Peninsular Malaysia was demonstrated in this study. In line with previous studies, *B. bovis* was genetically diverse, as evidenced by the identification of six genotypes based on molecular methods. MLST data shows the identification of new sequence variants of *B. bovis* which are distinct from those reported in other geographical regions. A new lineage (IIa) is proposed for a single isolate of *B. bovis* obtained from a dairy cow. The vectorial potential of *H. bispinosa* ticks of *B. bovis* between cattle warrants further investigation. Additionally, the role of *B. bovis* as a zoonotic infectious agent should be further explored, in view of the fact that humans may have direct exposure to *B. bovis* during handling of infected animals (slaughtering and evisceration process) and through bites of hematophagous arthropods in the farm.

### Availability of supporting data

The data set(s) supporting the results of this article is included within the article. The sequences derived from this study have been deposited in the GenBank database: *gltA* (GenBank accession no.:KP230412-KP230414), ITS (GenBank accession no.: KP230415-KP230417), and *rpoB* (GenBank accession no.: KP230418-KP230422). New sequence types from the MLST analysis: KR733181, KR733182-KR733184, KR733185-KR733186, KR733187-KR733189, KR733190-KR733191, KR733192-KR733195, KR733196, KR733197-KR733121 for *ftsZ*, *gltA*, *groEL*, *nuoG*, *ribC*, *rpoB*, *ssrA* and ITS, respectively.

## Abbreviations

ERIC-PCR: Enterobacterial repetitive intergenic consensus polymerase chain reaction
MLST: Multilocus Sequence Typing
